# There’s no place like home: optimizing the antepartum inpatient experience

**DOI:** 10.1186/s40748-024-00185-5

**Published:** 2024-08-01

**Authors:** Ashley M. Hesson, Kavya Davuluri, C. Kenzie Corbin, Anna M. Rujan, Deborah R. Berman

**Affiliations:** 1 Department of Obstetrics & Gynecology, Division of Maternal Fetal Medicine, University of Michigan, 1500 East Medical Center Dr, Ann Arbor, MI 48109 USA; 2 Department of Obstetrics & Gynecology, Northwestern University, Chicago, IL USA

**Keywords:** Hospitalization, Cost, Disparities, Patient-provider communication, Satisfaction

## Abstract

**Background:**

To characterize the demographics of a modern hospitalized antepartum population, compare the morbidities of this subset to national morbidity trends, and identify predictors of satisfaction during hospitalization to inform opportunities to enhance equitable antepartum care.

**Methods:**

Pregnant people admitted to the antepartum service of a large university hospital between 2011 and 2019 were surveyed about their hospitalization, pregnancy outcomes, provider interactions, perceived needs, and resource use. Multiple correspondence analysis was used to group patient responses based on latent relationships among demographic, medical, and psychosocial variables. Multivariate analyses were conducted to identify predictors of patient experience rating. Patient free text responses were qualitatively analyzed for common themes.

**Results:**

Of 740 pregnant people invited to participate, 298 surveys met criteria for analysis. 25.2% of these pregnant people identified as non-white and 20.8% were admitted for the management of a chronic medical condition. Patient responses clustered into three representative groups: (1) working pregnant people facing resource limitations, (2) first-time pregnant people with college educations, and (3) pregnant people with medical problems and limited partner support. The mean overall patient admission experience rating was 8.4 ± 1.7 out of 10. Variables represented in Cluster 1 (working and resource limitations) were associated with lower patient experience rating (*p* < 0.01). There was no significant variation in experience rating with indication for admission (*P* = 0.14) or outcome of the pregnancy (*P* = 0.32). Conversely, feeling supported by partners (*P* < 0.01) and providers (*P* < 0.01) directly correlated with a better experience.

**Conclusion:**

Black pregnant people and those with chronic medical conditions are overrepresented in this antepartum population when compared to the demographics of those not requiring hospitalization in pregnancy, where these groups also have higher rates of maternal morbidity and mortality at the national level. The most important contributors to patients’ satisfaction with their antepartum experience are feeling listened to by providers and supported by partners. Improving patient-provider communication and partner engagement during antepartum admissions should be a focus of inpatient high-risk obstetric care.

**Supplementary Information:**

The online version contains supplementary material available at 10.1186/s40748-024-00185-5.

## Introduction

Black pregnant people are the most likely to experience morbidity and mortality when hospitalized prior to delivery [1]. The medical and social drivers of this fact remain poorly understood however, largely due to inadequate research on the experience of pregnant people hospitalized in the antepartum period. The most recent review of the antepartum hospitalized population’s demographics is outdated, representing the late 1990s and early 2000s. At that time, antepartum hospitalizations occurred in 12.8 per 100 deliveries with higher rates for Black pregnant people and pregnant people without private insurance [2]. These admissions were largely brief, less than 72-h hospitalizations for preterm labor evaluation (likely representing the 48–72 h course of betamethasone administration). The psychosocial implications of these stays are different from those of prolonged stays over several weeks. Longer stays have a greater potential to affect patients’ mental health, especially for those who are under-resourced, as they worry about their financial and social responsibilities alongside concerns about their fetus’ and their own health [3, 4]. Data are lacking for more recent cohorts and longer-term stays. Furthermore, there is no large-scale research on the psychosocial aspects of patients’ experiences; insight into these facets of antepartum care may identify actionable means by which to improve both biomedical and psychosocial outcomes.

It is important to understand the composition of the modern antepartum patient population and what factors influence the hospitalization experience to identify opportunities for strategic interventions that empower biopsychosocial health and combat disparities. Thus, we pose the following questions in this pilot research study:

1. What are the characteristics of pregnant people hospitalized in the antepartum unit of a large tertiary care center?
2. How do the morbidities experienced by hospitalized antepartum patients compare to national trends in maternal morbidity and mortality?
3. What predicts overall satisfaction with an antepartum stay across this population and within sub-groups?

## Materials and methods

Patients were recruited for this survey-based cross-sectional study following their inpatient stay at a large, Midwestern university hospital that performs over 5000 deliveries per year. Its catchment area includes the local community and surrounding counties across multiple states, reflecting its status as a referral center for complex maternal–fetal management. On average, 72.7 ± 33.0 patients per year met the study criteria below during the eight-year study period.

Pregnant people hospitalized between 2017 and 2019 were surveyed on a rolling basis. Due to low participation, patients meeting criteria for admissions occurring from 2011–2016 were retrospectively invited to participate in the same survey. Sensitivity analyses demonstrated the comparability of these differently sampled cohorts (Supplement 1), thus the two time periods are integrated.

Obstetric patients at least 18 years of age who were admitted to this hospital’s antepartum unit for at least 96 h were considered eligible for participation. The 96-h cutoff was selected: 1) to exclude individuals admitted for short term stays with anticipated endpoints of 48–72 h, such as admission for a betamethasone course and 2) to exceed the mean total length of stay for high-risk patients at the study institution.

Patients were initially contacted via mail or follow-up phone call. Written consent was obtained from all participants. Patients with multiple antepartum stays were only surveyed once. Patients admitted for at least 96 h but who did not spend that time as antepartum patients (e.g., long induction of labor, complicated postpartum course) were excluded from analysis, as were those with incomplete surveys. The study was approved by the institutional review board (HUM00114580).

The survey instrument was provided as a paper copy that could be physically filled out and returned with an identical electronic version available upon request. It contained 36 questions about demographics, hospitalization, and social relationships. Respondents were also asked to indicate their satisfaction regarding this hospital experience on a 1–10 scale, with 10 being the best experience, with an opportunity for free response. All of the survey items were developed and refined through multidisciplinary consensus review by experts in Maternal Fetal Medicine, Psychiatry, and survey methodology.

Chart review was used to supplement survey responses with data regarding pregnancy number, outcome, comorbidities, and complications. Where available, demographic information was also inputted.

The analysis was conducted using a mixed-methods approach, with descriptive statistics for demographics, statistical modeling for categorical data, and thematic analysis for free response data. Within the statistical modeling, Multiple Correspondence Analysis (MCA) [5] was performed to identify participant traits that tended to co-occur, representing patterns or clusters. Variables with significantly different satisfaction ratings across their levels were included in a multivariate analysis predicting patient satisfaction. All quantitative analyses were conducted in R (The R Foundation, Vienna, Austria). Thematic qualitative analysis of the free response entries was performed by a trained author.

## Results

A total of 740 pregnant people were invited to participate, and 408 surveys were returned (55.1% response rate). Of these, 298 met the criteria for analysis. Demographics and fetal outcomes for the sample are given in Table 1. Most of the participants self-identified as employed (*n* = 193, 64.8%). Just under half reported having a college degree (*n* = 131, 44.0%). Approximately one quarter of the sample identified as non-partnered (*n* = 63, 21.1%) and as a non-white race (*n* = 75, 25.2%), with most of these individuals identifying as Black.

**Table 1:** Maternal demographics and fetal outcomes for the 298 analyzed antepartum stays. Basic demographics are provided for the 206 non-respondent antepartum patients

	Antepartum survey respondents (*n* = 298)	Antepartum non-respondents (*n* = 206)	
Parameter	n (%) or mean ± SD		*P*
Demographics			
Age, years	30.7 ± 5.48	29.2 ± 6.04	< 0.01*
Nulliparity	119 (39.9)	74 (35.9)	0.058
Employed	193 (64.8)	UA	
College-educated	131 (44.0)	UA	
Non-white race	75 (25.2)	66 (32.0)	0.155
Black	43 (14.4)	33 (16.0)	
Other	32 (10.7)	33 (16.0)	
Reason for admission			
PPROM	69 (23.2)	64 (31.1)	< 0.01*
Maternal co-morbidity	62 (20.8)	29 (14.1)	
Hypertensive disorder of pregnancy	56 (18.8)	30 (14.6)	
Non-reassuring antenatal testing	42 (14.1)	17 (8.3)	
Preterm labor	39 (13.1)	44 (21.4)	
Bleeding	30 (10.1)	22 (10.7)	
Outcomes			
IUFD	14 (4.7)	UA	
Postnatal demise	25 (8.3)	UA	
Prematurity	251 (84.2)	UA	
Gestational age at delivery, days	224.9 ± 32.1	225.3 ± 29.5	0.907
Major morbidity in survivors	18 (6.9)	UA	

*IUFD* Intrauterine fetal demise, *NICU* Neonatal intensive care unit, PPROM Preterm prelabor rupture of membranes, *UA* Unavailable

*P<0.05

Preterm prelabor rupture of membranes (PPROM; *n* = 69, 23.2%) and maternal chronic medical conditions (*n* = 62, 20.8%) were the most common indications for admission. The mean gestational age at delivery was 32 completed weeks. A few patients 39 (13.1%) reported offspring demise as the outcome of the pregnancy. Of the surviving children, 6.9% (*n* = 18) were described by participants as having “significant” physical or mental health needs following discharge.

The average duration of participant stay was 15.5 days (range 4–96 days). The mean experience rating was 8.4 ± 1.7, which did not vary significantly by delivery year (*P* = 0.90) or race (*P* = 0.91). Over one third of those surveyed expressed difficulty maintaining their residences while admitted (*n* = 115, 38.6%); 16.4% (*n* = 49) described difficulty managing school or work, and 25.8% of participants (*n* = 77) reported experiencing significant hardship due to the costs of admission. Black pregnant people were more likely to experience financial hardships (*P* < 0.01).

Most pregnant people felt their partners (*n* = 271, 90.9%) and providers (*n* = 287, 96.3%) were at least somewhat helpful during their stay; 85.9% (*n* = 256) of respondents felt that their provider listened to them a majority of the time or more. There was no difference in the distribution of listening rating based on patient race (*P* = 0.97).

### Patient clusters

**Fig. 1:**
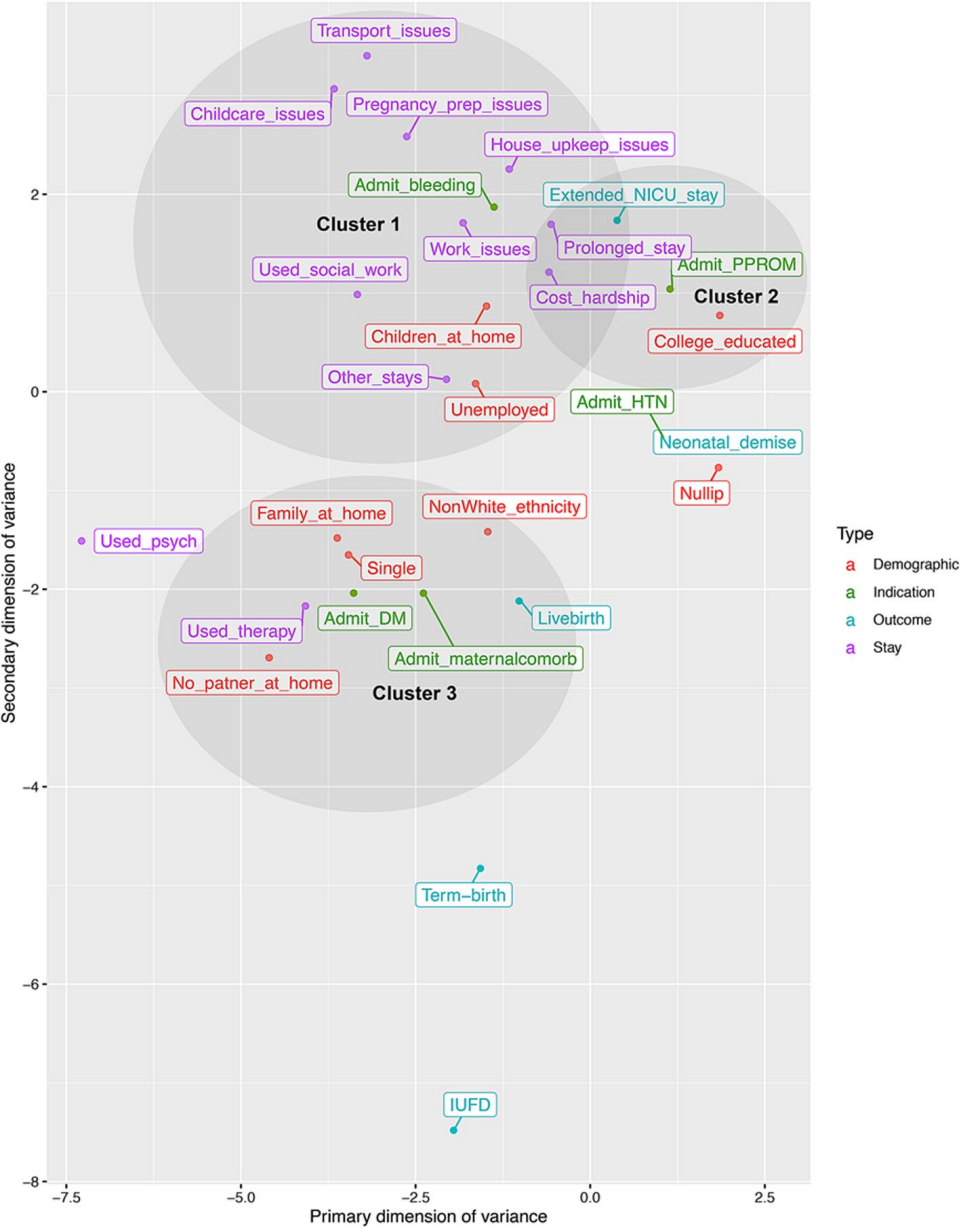
Biplot of antepartum characteristics. A biplot representation of the two-dimensional MCA performed for categorical variables abstracted from patient survey responses with scaled inertias > 1. Clusters 1, 2, and 3 (interpreted in the text) are denoted by shaded ovoids and labeled

Three clusters of participant characteristics emerged empirically in the MC*A* analysis: 1) Working pregnant people facing resource limitations, 2) First time pregnant people with college educations, and 3) Pregnant people with medical problems and limited partner support. We discuss each of these three clusters, summarizing the results of the MCA (Fig. 1) integrated with the findings of the post-hoc qualitative analysis.

#### Cluster 1: Working pregnant people facing resource limitations

Cluster 1-like pregnant people self-identify as having children at home and being employed; concerns regarding disruption of their family dynamic, missed time at work, and financial strain define their experience of long-term antenatal hospitalization. They are the most likely to use Social Work resources, as reflected in the inclusion of Social Work use in Cluster 1 (Fig. 1). They seek support for childcare, transport, and lost time at work:

“If we would have received more support our stress level may have been lower. Perhaps my blood pressure would have stabilized.”

Beyond their logistic needs however, these patients expressed feelings of guilt for their absence and emphasized the effects of their hospitalization on their partners, children, and support networks:

“My husband, without notice, was thrusted into single parenthood. He and the kids needed to quickly develop new routines and comfort zones.”

“I was gone, my husband had to take over all my daily duties while continuing to work his job. He was worried and alone.”

They also acknowledged the importance to their physical and mental wellbeing of having family and friends engaged in their hospitalization. Patients who did not report a need for additional support services in the hospital often cited the contributions of their families to their social, emotional, and logistic needs. Their qualitative comments retell narratives of their time in the hospital as a community experience, one that is more focused on social integration than on medical events or recommendations for improvement:

“My partner, family and close friends provided a lot of support. They visited often and my partner was there every day whenever he was not at work.”

#### Cluster 2: First time pregnant people with college educations

Participants represented by this cluster self-identify as having some college experience, which is then reflected in an intellectualized experience of long-term hospitalization in pregnancy. Cluster 2-like patients center their experience around their diagnosis. They are most likely to be admitted with PPROM or a hypertensive disorder. These patients have the highest rate of neonatal demise. They report a desire for more information to help contextualize these difficult experiences, both in terms of their immediate implications and their long term physical and emotional sequelae (e.g., “I felt unprepared for what I endured as a result of preeclampsia.”). They equate feeling informed to a positive experience, and a lack of information (either passively acquired or actively requested) as unacceptable:

“The doctors could have communicated better. We had many questions that were not answered.”

“My situation and diagnosis were unclear and should have been explained sooner.”

Cluster 2-like patients describe feelings of isolation, fear, and loneliness along with regret for not requesting assistance. One participant described feeling:

“Totally overwhelmed and scared during my pregnancy. During my hospitalizations I was so afraid of dying. I regret not reaching out for more psychosocial support.”

Collectively, Cluster 2-like individuals highlight a discordance between their current post-partum understanding of their pregnancy events and their state of being while hospitalized.

#### Cluster 3: Pregnant people with medical problems and limited partner support

Cluster 3-like patients report being single and living with family members. They are the most likely to identify as non-white race and are most often admitted for management of chronic conditions. They were the most frequent users of therapy resources during their stay. Cluster 3-like patients, those without partner support or with long-term medical conditions, focused on the management of these conditions. They tend to anchor themselves on objective data such as blood pressures or blood glucose values. They express a sense of empowerment, both in their medical care and in identifying their own needs for support resources:

“Managing my blood sugars successfully while pregnant would not have been possible if I had not taken an active role and primary responsibility for my care.”

Cluster 3-like patients had clear perspectives on their care that were formulated at the time of their hospitalization. They often referenced taking ownership of their physical and mental health needs:

“I have much respect [for] all the doctors but sometimes they are so 'by the book'/ I wanted to scream. There is no 'cookie cutter' pregnancy. I was getting drained being asked what my sugar numbers were!”

“The experience was traumatic- needed support from therapist. No family support”

### Patient experience

Categorical analysis showed experience rating was not associated with pregnancy outcome (*P* = 0.91), indication for admission (*P* = 0.32), relationship status (*P* = 0.40), multiparity (*P* = 0.47), college experience (*P* = 0.80), employment (*P* = 0.59), or a particular racial background (*P* = 0.99). However, concerns over the costs of their hospitalization (*P* < 0.01) and difficulty arranging upkeep of their home (*P* < 0.01), traits associated with Cluster 1, were associated with lower experience ratings. Conversely, increasing patient age (*P* < 0.01), partner engagement in care (*P* < 0.01), and provider communication skills–especially listening skills (*P* < 0.01)–were positively correlated with patient experience rating.

In multiple regression modeling of patient experience rating as a function of cost concerns, patient age, partner engagement, and provider listening rating, only partner engagement rating and provider listening rating were retained as significant predictors (*P* = 0.01 and *P* < 0.01 respectively). The model details are given in Supplement 2.

Qualitative analysis of participant comments underlined the importance of provider communication and partner support. Negative comments pertaining to providers focused exclusively on communication. Conversely, patients described partner support in the form of bedside companionship as well as management of household affairs (see Patient Clusters).

## Discussion

The demographics and outcomes observed in our antepartum sample suggest that non-white, specifically Black, pregnant people continue to bear a disproportionate amount of the burden of extended hospitalization, consistent with earlier literature. Though most participants were white, there was a higher proportion of non-white individuals in the antepartum sample (25.2%) than the 19–20% of births occurring to non-white people in the region [6]. Furthermore, consistent with existing literature, non-white race clustered with having high-risk, chronic medical conditions requiring hospitalization [7, 8] and needing therapy resources in the present analysis [9, 10]. In our sample, they also reported significantly greater financial distress from admission.

We find that the average antepartum stay is longer than two weeks, considerably longer than the three day mean hospitalization length cited in prior work [2]. Accordingly, PPROM and maternal medical co-morbidities were the most common indications for admission; these require close monitoring for extended periods of time. This is likely due in part to our purposeful exclusion of very brief admissions, consistent with our aim of studying longer admissions to identify the unique burdens associated with them. Per our patient clusters and qualitative analysis, pregnant people’s individual struggles vary along with their diagnoses, backgrounds, and support systems.

The only non-modifiable factor predicting satisfaction with an extended hospitalization was patient age. The three modifiable factors that predicted improved patients’ experience ratings were partner engagement, provider listening, and the presence/absence of cost concerns.

The engagement of a pregnant person’s partner, support person, or supportive unit in their care is profoundly understudied. Given the nature of obstetric care, the focus is inherently placed upon the pregnant person and their fetus more than the surrounding social context. However, it is crucial to note strategies to help this vulnerable patient population, including partners and support people in obstetric care include generous visitor policies, alternative housing arrangements [11]. and interventions aimed at empowering social support, such as talk or music therapy. Further research is warranted in this domain.

Patient-provider communication in inpatient obstetrics also lacks a robust evidence base. Studies have considered the use of shared decision-making models in determining mode of delivery [12] and for determining goals of care in the peri-viable period [13], but these touchpoint decisions represent few of the discussions that need to take place in a lengthy antepartum admission. For example, providers seldom engage in deep, useful conversations regarding financial hardship [14], though patients would prefer to engage in such conversations with their providers [15]. This is notable as financial distress and difficulty arranging care for dependents were prevalent in our sample, consistent with prior literature [2]. Moreover, pregnant people of color and socioeconomically disadvantaged pregnant people are less likely to report active participation in decision-making processes during their admissions [12].

Though this pilot study identifies actionable foci for improving the antepartum experience, there are limitations. Our study draws from a single regional care center that may not be representative of the antepartum population in other settings. Furthermore, though our recall-based survey methodology allowed for quantitation over a large sample, the period from delivery to survey response was variable and, in some cases, spanned several years. We also had a substantial number of non-responders for whom detailed comparative data was unavailable, thus introducing the possibility of response bias. Lastly, this study focused specifically on patient-physician interaction as opposed to patient interaction with nurses and support staff; these interactions deserve specific attention especially in light of the importance of provider communication highlighted in this study.

Despite these limitations, our study highlights how antepartum admissions reflect larger trends in maternal–fetal morbidity, where under-resourced, non-white pregnant people suffer disproportionately. Furthermore, patients’ perspectives highlight an opportunity for intervention– they point to provider communication and partner support as having a potentially modifiable and significant impact on their inpatient experience. Techniques for facilitating patient-provider engagement can be applied to empower those at highest risk of psychosocial distress during their hospitalization [16]. Strategies for improving partner support are needed to better inform intervention.

## Conclusion

Our study of pregnant people admitted to an antepartum service draws attention to the diversity of this group while also highlighting their shared prioritization of effective provider communication and social support.

### Supplementary Information

Supplementary Material 1. Sensitivity analysis comparing length of stay and satisfaction ratings between those responding most proximally and most distally to their admission. The most proximal were defined as either: 1) the 10% (*N*=29) responding with the shortest interval between their admission and their survey return (in the “by-interval” analysis) or 2) the latter portion of the study period (2017-2019, in the “by-year” analysis). The most distal were defined as either: 1) the 10% (*n*=29) responding with the longest interval between their admission and their survey return (in the“by-interval” analysis) or 2) the earlier portion of the study period (2011-2016, in the “by-year” analysis). The mean interval between delivery and survey return was 117.0±82.6 weeks.

## Data Availability

The datasets used and/or analyzed during the current study are available from the corresponding author on reasonable request.
