# Supplementary material for: There’s no place like home: optimizing the antepartum inpatient experience
Source: Matern Health Neonatol Perinatol. 2024 Aug 1;10:15. doi: 10.1186/s40748-024-00185-5 (PMC11293146; doi:10.1186/s40748-024-00185-5)
Supplement: Supplementary file 1 — Supplementary Material 1. Sensitivity analysis comparing length of stay and satisfaction ratings between those responding most proximally and most distally to their admission. The most proximal were defined as either: 1) the 10% (N=29) responding with the shortest interval between their admission and their survey return (in the “by-interval” analysis) or 2) the latter portion of the study period (2017-2019, in the “by-year” analysis). The most distal were defined as either: 1) the 10% (n=29) responding with the longest interval between their admission and their survey return (in the“by-interval” analysis) or 2) the earlier portion of the study period (2011-2016, in the “by-year” analysis). The mean interval between delivery and survey return was 117.0±82.6 weeks. [file 40748_2024_185_MOESM1_ESM.docx]

**Supplement 1.** Sensitivity analysis comparing length of stay and satisfaction ratings between those responding most proximally and most distally to their admission. The most proximal were defined as either: 1) the 10% (N=29) responding with the shortest interval between their admission and their survey return (in the “by-interval” analysis) or 2) the latter portion of the study period (2017-2019, in the “by-year” analysis). The most distal were defined as either: 1) the 10% (n=29) responding with the longest interval between their admission and their survey return (in the “by-interval” analysis) or 2) the earlier portion of the study period (2011-2016, in the “by-year” analysis). The mean interval between delivery and survey return was 117.0±82.6 weeks.

| Parameter | Proximal group mean | | Distal group mean | | P | |  |
| --- | --- | --- | --- | --- | --- | --- | --- |
| Analysis | by-interval | by-year | by-interval | by-year | by-interval | by-year | |
| Length of stay (days) | 13.75 | 16.01 | 16.67 | 14.23 | 0.447 | 0.339 | |
| Satisfaction score | 8.46 | 8.29 | 8.14 | 8.48 | 0.567 | 0.416 | |

**Supplement 2.**  Multiple regression analysis predicting increasing patient experience rating. Multiple R^2^ for the model is 0.314.

| Parameter | Value | Estimate | Standard error | P |
| --- | --- | --- | --- | --- |
| Maternal age | Continuous | 0.0278 | 0.0157 | 0.08 |
| Cost concerns | Present | -0.155 | 0.208 | 0.46 |
| Partner engagement rating | Continuous | -0.196 | 0.0734 | < 0.01 |
| Provider listening rating | Continuous | 0.641 | 0.0644 | < 0.01 |
